# Evolutionary Systems Biology

**DOI:** 10.2174/138920211797248592

**Published:** 2011-09

**Authors:** Jianying Gu

Systems biology has a long history as a theoretical framework in biology, positing that cellular systems possess emergent properties that can only be explained by the interactions among the components of an organism rather than by any individual component in itself. With the advent of the genomic era, data sets encompassing systems-wide measurements of gene complements, transcription, and protein expression are becoming available. Thoughtful integration of these data can lead to a systems-level understanding of the components and dynamics of cellular machineries and the development of practical methods for inferring and constructing network models of these interactions is well under way. As the creation of plausible network models of cellular systems becomes possible, comparison of systems in an evolutionary light promises to reveal the evolutionary forces that shape these systems. Systems are dynamic and selectable, that is, they respond to pressures brought to bear by changes in their environment. Therefore, the application of the methods of evolutionary biology within a systems biology framework can aid in identifying the concerted changes that drive the emergence of new phenotypes. A better understanding of systems evolution will greatly advance our knowledge about the complexity of gene regulation and help identify new therapeutic targets for effective treatment of various diseases.

This special issue of *Current Genomics* is intended to provide a forum for researchers to share their experiences on all aspects of genome and systems evolution. This issue includes a review article discussing about the proteomics techniques that are capable of generating high quality data for systems biology research: Chen *et al.* reviewed recent advances in proteolysis, a key procedure prior to mass spectrometry and peptide mapping in proteomic studies of gene function. They showed that the proteolysis strategies can be accelerated by various electromagnetic waves, such as microwave-accelerated protein digestion, infrared-assisted proteolysis, ultraviolet-enhanced protein digestion, laser-assisted proteolysis, and future prospects. This issue also includes two review articles with a special emphasis on the systems biology of bacteria, ideal model organisms for evolutionary analyses of genome plasticity and adaptive phenotypes. Tang's review is focused on microbial metabolomics, which involves fast, high throughput characterization of the metabolite complements of a microorganism, and thus summarizes the global outcome of the interplay between the developmental processes and the environment. Selected topics illustrate the impact of metabolomics on the understanding of systems microbiology. Zhou *et al.* provide an overview of the recent advances in *Streptomyces* biology that have been driven by high throughput technologies. The omics based studies have revolutionized the understanding of system control and regulation dynamics of this group of bacterial of medicinal and industrial importance. Evolutionary systems biology approaches have also been applied to biomedical studies. Cai *et al.* provide a phylogenomic overview of the origin, distribution, diversity, and evolution of malarial proteases, a class of promising therapeutic targets for combating this devastating infectious disease that is responsible for over 1 million deaths yearly. Klemcke *et al.* introduce a novel physiogenomic approach to the study of hemorraghic shock, a major cause of death throughout the world. Their findings in heritable quantitative trait loci, as well as potential epigenetic mechanisms that might influence survival time after severe hemorrhage, may lead to the improvement in survival of traumatic hemorrhage and provide knowledge of genetically-informed personalized treatments.

Overall, these reviews provide a fascinating survey of the combination of systems thinking and evolutionary analysis across the biological spectrum. As modeling approaches improve and as more data from more species become available, the study of systems evolution will clarify the links among the genotype, the phenotype and the selective pressures arising from the organism’s place in the environment. As an emerging field in the post genomic era, evolutionary systems biology promises to make profound contributions to our understanding of life.

